# The NAC Transcription Factor *ANAC087* Induces Aerial Rosette Development and Leaf Senescence in Arabidopsis

**DOI:** 10.3389/fpls.2022.818107

**Published:** 2022-02-23

**Authors:** Brenda Yazmín Vargas-Hernández, Leandro Núñez-Muñoz, Berenice Calderón-Pérez, Beatriz Xoconostle-Cázares, Roberto Ruiz-Medrano

**Affiliations:** Departamento de Biotecnología y Bioingeniería, Laboratorio de Biología Molecular de Plantas, Centro de Investigación y de Estudios Avanzados del Instituto Politécnico Nacional, Mexico City, Mexico

**Keywords:** Arabidopsis, *ANAC087*, phloem, aerial rosette, senescence

## Abstract

CmNACP1 mRNA has been shown to move long distance through the phloem in *Cucurbita maxima* (pumpkin) and through a graft junction. Whereas the phloem transport of several different mRNAs has been documented in other systems as well, its function remains, for most of these RNAs, largely unknown. To gain insight into the possible role of these RNAs, we searched for the closest homologs of *CmNACP1* in Arabidopsis, a model plant much more amenable for analysis. A phylogenetic approach using the predicted NAC domain indicated that ANAC059, ANAC092, ANAC079, ANAC100, ANAC046, and ANAC087 form a single clade with CmNACP1. In the present work, we analyzed the possible function of the *ANAC087* gene in more detail. The promoter region of this gene directed expression in the vasculature, and also in trichomes, stem, apexes, and developing flowers which supports the notion that *ANAC087* and *CmNACP1* are orthologs. Overexpression of the *ANAC087* gene induced increased branching in inflorescence stem, and also development of ectopic or aerial rosettes in T1 and T2 plants. Furthermore, overexpression of *ANAC087* leads to accelerated leaf senescence in 44 days post-germination (dpg). Interestingly, a similar phenotype was observed in plants expressing the *ANAC087* gene upstream region, also showing an increase in *ANAC087* transcript levels. Finally, the results shown in this work indicate a role for *ANAC087* in leaf senescence and also in rosette development.

## Introduction

The NAC domain (for Petunia NAM, and Arabidopsis ATAF1, ATAF2 and CUC2) family of transcription factors is ubiquitous in plants playing diverse important roles in development and response to external stimuli. These include establishing boundaries and organ primordia formation in the shoot apical meristem (SAM), phloem and xylem differentiation, response to salt stress, and pathogen-induced cell death (Souer et al., 1996; Aida et al., 1997; Olsen et al., 2005; Furuta et al., 2014; Nieminen et al., 2015; Podzimska-Sroka et al., 2015; Oda-Yamamizo et al., 2016; Lee et al., 2017). A role in nuclear-organellar signaling in response to stress has also been established for these proteins (De Clercq et al., 2013; Pascual et al., 2021). Several taxa appear to have an inordinate number of NAC domain protein genes, such as Arabidopsis, in which at least 100 genes have been found, which suggest functional redundancy (Ooka et al., 2003). Certain NAC domain proteins have been shown to enhance senescence, in some cases through binding to conserved sequences in the upstream region of target genes, for example, those involved in the degradation of chlorophyll (Guo and Gan, 2006; Balazadeh et al., 2010; Kim et al., 2016).

This protein family has been analyzed in detail mostly in Arabidopsis; less is known regarding their function in other species. However, a large proportion of these genes in crop plants (close to one-third) appear to be associated with senescence, based on transcriptomic analyses (Podzimska-Sroka et al., 2015). Senescence is an active process driven by different signals such as hormones, sugars, reactive oxygen species (ROS), and calcium (Bresson et al., 2018). Several families of transcription factors regulate this process, particularly NAC and WRKY transcription, based initially on their induction at the onset of senescence (Guo et al., 2004; Lin and Wu, 2004; Buchanan-Wollaston et al., 2005; Balazadeh et al., 2008; Liu et al., 2010; Breeze et al., 2011; Li et al., 2012). Many of these genes are positive regulators of senescence. For example, *ANAC092/ORESARA1* (*ORE1*) affects the expression of many genes involved in nutrient relocation processes, cell wall modifications, and hormone metabolism during senescence development, and also in response to external stimuli (salt stress- or ethylene and salicylic acid-induced senescence; Balazadeh et al., 2010; Wang et al., 2021). Also, *ANAC059 (ORS1)* induces senescence in response to H_2_O_2_ (Balazadeh et al., 2011); the induction of *ANAC055*, *ANAC029/Arabidopsis NAC-LIKE Activated by AP3/PI (AtNAP)*, *ANAC16*, and *ATAF1* correlates with the start of senescence (Guo and Gan, 2006; Hickman et al., 2013; Kim et al., 2013; Garapati et al., 2015). Other *NAC* genes are negative regulators of leaf senescence, for example, *ANAC042 (JUB1)* and *ANAC083 (VNI2)* (Yang et al., 2011; Wu et al., 2012).

The pumpkin *CmNACP1* mRNA accumulates in mature, functional SE and was found to move long distance across a graft union into the shoot apex of a cucumber heterograft (Ruiz-Medrano et al., 1999). Several other transcripts’ coding for NAC domain proteins has been found in phloem sap transcriptomes, which suggest a non-cell autonomous function of this gene (Ruiz-Medrano et al., 2007; Rodriguez-Medina et al., 2011). The accumulation of CmNACP1 in the shoot apex suggests a role similar to NAM in Petunia and CUC2 in Arabidopsis (formation of apical meristem and separation between the meristem and organ primordia; Souer et al., 1996; Aida et al., 1997). However, one of its closest homologs in terms of sequence similarity of the NAC domain was found to be the NAC protein senU5 from tomato, induced during leaf senescence, which suggests an additional role for *CmNACP1* in this process (John et al., 1997; Ruiz-Medrano et al., 1999). In this work, the Arabidopsis homologs of CmNACP1 were searched for analyzing their function in more detail.

We found that CmNACP1 forms a clade with ANAC087 (At5g18270) and ANAC046 (At3g04060), among other NAC proteins related to induction of cell death and senescence. Based on the sequence similarity to CmNACP1, ANAC087 is one of its closest homologs. This gene was studied in a more detailed fashion. The results presented herein indicate that *ANAC087* is expressed in the shoot apex, roots, and vascular tissue, inducing senescence in rosette leaves, and also emergence of aerial rosettes in T1 and T2 plants; a similar phenotype was observed in plants expressing the promoter region of this gene.

## Materials and Methods

### Phylogenetic Analysis

A cladistic analysis was performed in Arabidopsis to find the homologous genes of CmNACP1; for this purpose, we used the conserved N-terminal domain of the NAC protein family as a bait. First, a BLAST alignment was carried out with the CmNACP1 NAC domain reported in UniProt Knowledgebase^1^ to select similar sequences in Arabidopsis. All sequences obtain displaying homology were aligned by MUSCLE multiple sequence alignment software. The phylogenetic tree was constructed with MEGA7^2^ using the neighbor-joining (NJ) method and bootstrap test carried out with 1,000 iterations.

### Plant Material and Growth Conditions

Seeds of *Arabidopsis thaliana* ecotype columbia-0 were sown on sterilized soil (agrolite-peat-soil, 1:2:2) and grown in a controlled climate chamber (16-h light/8-h dark) at 22 ± 2°C. For transgenic plants, seeds were germinated in soil and then in the seedling stage were sprayed every 6 days with the herbicide containing ammonium glufosinate at the recommended dilution of 1:6000 (Invictus, Allister; Zapopan, Mexico) for one month. Fifteen plants per independent Arabidopsis line were grown in pots containing sterilized soil in the controlled climate chamber AR715 (Percival; Perry, Iowa). The phenotype of transformed plants was assessed by measuring branch number and parameters related to leaf senescence.

### Plasmid Constructs

To analyze and study *ANAC087*, we obtained two genetic constructs with different regions of the *ANAC87* gene (GenBank accession number NM_121832.4). The *ANAC087* open reading frame (ORF, 1005 bp) was cloned into a binary vector (pB7FWG2,0; Plant Genetic Systems, Ghent, Belgium) under the control of the CaMV 35S promoter to analyze the effect of its overexpression on plant development. The analysis of *ANAC087* promoter region was carried out using 3.0 kb upstream region of the ORF, which includes the 5′ untranslated region. For this purpose, the promoter was subcloned into the pBGWFS7,0 Gateway vector (Supplementary Figure 1). To obtain recombinant plasmids, the following procedures were carried out. First, total RNA and genomic DNA were extracted from rosette leaves of *A. thaliana* wild type using Direct-zol™ RNA Miniprep Kit (Zymo Research, Irvine, United States) and CTAB method (Weigel and Glazebrook, 2002), respectively. The primers designed to amplify the promoter region and the ORF are reported in Supplementary Table 1. To obtain the ORF of *ANAC087*, cDNA was synthesized employing SuperScript III First-Strand Synthesis System (Invitrogen, La Jolla, CA, United States) using 50 ng of total RNA, oligo (dT) and SMAGGG primers at 10 μM each, a mixture of dNTPs 10 mM (New England Biolabs, Beverly, MA, United States), and sterile distilled water to a final volume of 13 μL. The reaction mixture without enzyme was incubated at 72°C for 10 min and kept on ice for 5 min. Then, 4 μL of 5′ buffer solution, 1 μL of DTT (0.1 M), 1 μL of RNase inhibitor, and 1 μL of SuperScript III RT enzyme (200 U/μL) were added to the reaction. The mixture was gently homogenized, incubated at 50°C for 1 h, and inactivated by incubation at 75°C for 15 min. Then, 1 μL of synthesized cDNA was used as a template to amplify *ANAC087* ORF with TaKaRa Ex Taq DNA Polymerase (Takara Bio Inc.) and specific primers, following the manufacturer’s instructions in a Biometra T1000 Thermocycler (Biometra AG, Germany). The same DNA polymerase was used to amplify 3.0 kb upstream sequence from the start codon of the *ANAC087* gene (promoter region) from genomic DNA. The amplicons obtained were cloned into the pCR8/GW/TOPO vector (Invitrogen, La Jolla, CA, United States) and sequenced to confirm the correct orientation. Then, the products were recombined into the corresponding binary vectors by Gateway LR reactions to generate the following genetic constructs: (1) *PromANAC087:GFP-GUS* construct or *PROM-ANAC087* and (2) *35S:ANAC087 ORF-GFP* construct or *OE-ANAC087*. Recombinant plasmids were analyzed by PCR and then introduced into *Agrobacterium tumefaciens* strain AGL1 by electroporation. Positive clones were cryopreserved and used for plant transformation.

### Plant Transformation

Transformation of Arabidopsis wild-type Co-1 ecotype was performed using the floral dip method (Clough and Bent, 1998) with some modifications: *A. tumefaciens* strain AGL1 harboring the recombinant plasmids was grown on plates containing three antibiotics (spectinomycin 50 mg/L, kanamycin 25 mg/L, and carbenicillin 50 mg/L). Then, immature flower buds were immersed in the bacterial suspensions; after the recovery of the plants, seeds were collected. Finally, transgenic plants were selected by herbicide resistance in soil with ammonium glufosinate, as previously described. T2- and T3-independent plant lines were used for further analysis.

### Histochemical Analysis

Samples of different tissues (rosette leaves, flowers, apexes, roots, stem, and petioles) were dissected from transgenic plants harboring *PROM ANAC087*:GFP-GUS construct. Histochemical detection of GUS activity was carried out according to Weigel and Glazebrook (2002). All tissues were covered with GUS solution and incubated at 37°C for 2 days and finally maintained in glycerol 50%. The tissues were visualized on a Nikon SMZ 745T stereoscope, and images were captured with a Nikon Digital Sight DS-U3 camera using NIS Elements D software.

### Phenotypic Analysis

Arabidopsis wild-type and transgenic lines harboring *PROM-ANAC087* and *OE-ANAC087* constructs were grown in soil under controlled conditions. Fifteen plants of each construct were analyzed, and images were captured at 20, 28, 36, and 44 days post-germination (dpg). Samples of rosette leaves were employed to quantify chlorophyll and anthocyanin content as described below.

### Measurement of Senescence Rate

Rosette leaves with 50% or more of yellowed, chlorotic leaf area were counted as senesced. The senescence rate was calculated by the ratio of rosette senescent leaves to the total number of leaves (Kim et al., 2018).

### Chlorophyll and Anthocyanin Extraction and Quantification

Rosette leaves at positions five and six were used for chlorophyll and anthocyanin quantification. Chlorophyll was extracted from rosette leaves at 44 dpg with phosphate buffer and 80% acetone, and absorbances were measured at 645 and 663 nm using spectrophotometer Smart Spec plus (Biorad, Hercules, CA, United States) following the protocol described by Arnon (1949). Anthocyanins were extracted with 45% methanol and 5% acetic acid, and absorbance measurement was carried out by triplicate at 530 and 657 nm (Nakata and Ohme-Takagi, 2014). Anthocyanins, total chlorophyll, chlorophyll a, and chlorophyll b concentrations were normalized to the fresh weight of each leaf.

### Quantitative Real-Time RT-PCR

Relative expression of the *ANAC087* transcript was determined in wild-type plants as well as in the transgenic lines for overexpression and promoter analysis. The quantification of genes associated with leaf senescence and relative *ANAC087* expression levels in all transgenic lines was performed using a set of primers within the 3′end of the *ANAC087* ORF, because this region is highly variable among the NAC gene family members (Ernst et al., 2004). Total RNA was extracted from different Arabidopsis tissues (root, rosette leaf, caulinar leaf, stem, flower, and siliques) using Direct-Zol™ RNA Miniprep kit (Zymo Research, United States) at 44 dpg. RNA extraction of each tissue was obtained from a pool of four plants. qRT-PCR with KAPA SYBR^®^ FAST One-Step kit was performed with an Applied Biosystems StepOnePlus™, following the manufacturer’s recommendations and 30 ng of total RNA in a 10 μL reaction. Specific primers for *ANAC087*, *UBQ10*, *RBCS1A*, *ORE1*, *SAG13*, and *ANAC046* were used (Supplementary Table 1). The *Ct* value for each product was determined by triplicate for each transgenic line. *UBQ10* was used as endogenous genes to normalize gene expression, and the comparative ΔΔ*Ct* method was used to determine relative transcript abundance (Livak and Schmittgen, 2001).

### Western Blot Analysis

Arabidopsis rosette leaves were collected to extract total proteins or nuclear proteins. For total protein extraction, leaves were mixed with extraction buffer (100 mM Tris–HCl *pH* = 6.8, 2% SDS, 200 mM DTT, 20% glycerol, and 0.5 mM PMSF). Extraction of nuclear proteins was performed employing the Plant Nuclei Isolation/Extraction Kit (CelLytic™ PN, Sigma-Merck, Darmstadt, Germany) following the manufacturer’s recommendations. Equal amounts of protein extracts were separated on SDS-polyacrylamide gels. The separated proteins were transferred onto Amersham Hybond-P PVDF Membrane (GE Healthcare, Chicago, United States) for 1 h at 100 volts. Membrane was blocked with TBST buffer, 0.1% Tween 20, and 5% skim milk for overnight at 4°C. Blots were incubated with antibody against ANAC087 (Genscript; Piscataway, NJ, United States) (1:2500) for 4 h at 4°C, washed, and incubated with a secondary antibody against rabbit IgG (whole molecule; Sigma-Merck) conjugated to goat peroxidase (1:10,000) for 2 h at room temperature. The washed blots were transferred to ECL Prime Western Blotting Detection Reagent (Pierce, Rockford, IL, United States) and then exposed to X-ray film.

## Results

### *ANAC059, ANAC092, ANAC079, ANAC0100, ANAC046, and ANAC087* Are the Arabidopsis Homologs of *CmNACP1*

**FIGURE 1:**
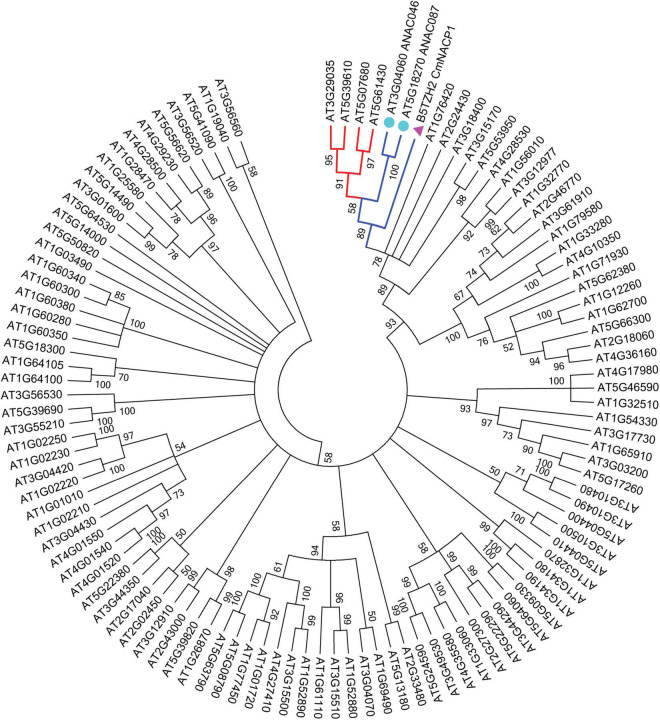
Phylogenetic analysis of NAC proteins from *A. thaliana* and CmNACP1. A total of 100 NACs from Arabidopsis were used to construct the neighbor-joining tree (NJ) based on NAC domain sequences. The phylogenetic tree was constructed by MEGA7 using a bootstrap value of 1,000 replicates. Homologous genes from *CmNACP1* (pink triangle) are denoted by (cyan circles) corresponding to *ANAC087* and *ANAC046*.

To identify the protein homologs for CmNACP1 in Arabidopsis, we performed a BLAST alignment with the CmNACP1 NAC domain reported in UniProt Knowledgebase (see text footnote 1), since the complete virtually translated sequences are of varying lengths, and sequences outside the NAC domain are highly divergent in members of the family, even within the same species. A total of 100 sequences were selected in Arabidopsis. To study the evolutionary relationship between the CmNACP1 and NAC proteins (NACs) from Arabidopsis, a phylogenetic tree was constructed from alignments of these NACs and CmNACP1 sequences (Figure 1). The results indicate that CmNACP1 homologs form a separate clade, which includes ANAC059 (At3g29035), ANAC092 (At5g39610), ANAC079 (At5g07680), ANAC100 (At5g61430), ANAC046 (At3g04060), and ANAC087 (At5g18270). The members of this clade are involved in leaf senescence, programmed cell death, and developmental regulation (Laufs et al., 2004; Raman et al., 2008; Balazadeh et al., 2010; Oda-Yamamizo et al., 2016; Lee et al., 2017; Huysmans et al., 2018), which suggests that the members of this clade are orthologs. The present analysis focused on ANAC087, since the sequence similarity with CmNACP1 considering the NAC domain and the whole protein are slightly higher than the rest of the members of this clade.

### *ANAC087* Is Expressed in the Vasculature, Shoot Apex, and Developing Flowers

**FIGURE 2:**
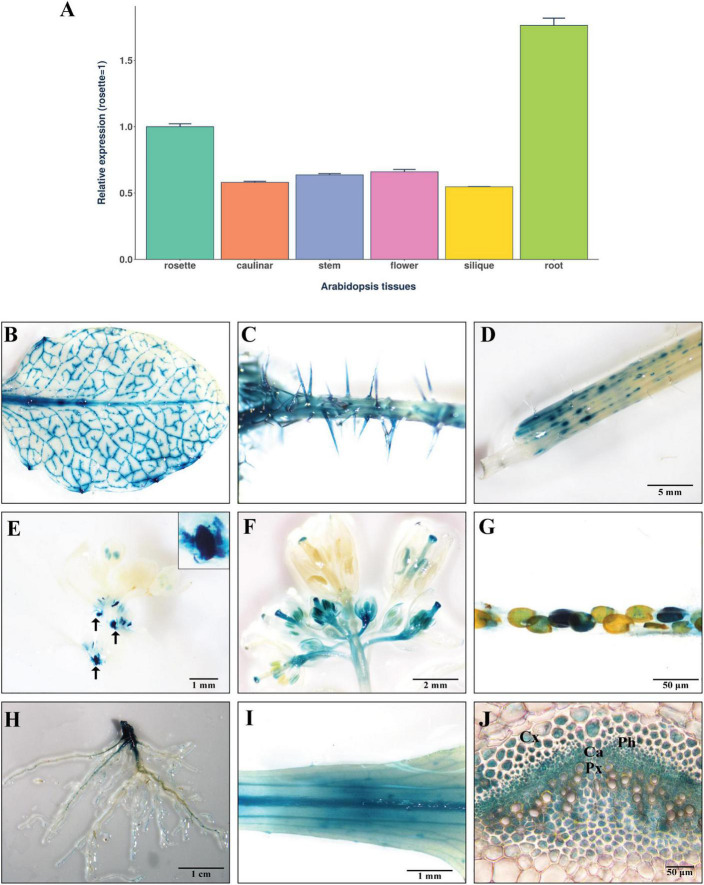
*ANAC087* expression and gene promoter activity in Arabidopsis. **(A)** Expression levels of *ANAC087* in rosette leaf, caulinar leaf, stem, flower, silique, and root in Arabidopsis wild-type plants. Relative expression was determined by a pool of four wild-type plants and three technical replicates. *A. thaliana* polyubiquitin 10 was used as endogenous gene, and relative expression levels were normalized to rosette leaf tissue equals to 1. **(B–I)** GUS histological staining in transgenic Arabidopsis lines containing p*ANAC087*:GUS construct in different tissues: **(B)** GUS activity detected in first-, second-, and third-order veins, **(C)** GUS detection in leaf petiole specifically in trichomes, **(D)** staining in sections of stem, **(E)** strong signal in shoot apices, **(F)** GUS detection in developing flowers but no signal was detected in open flowers, except in the papilla, **(G)** GUS signal in mature seeds, **(H)** main root and weaker GUS activity in lateral root, **(I)** detection of *ANAC087* promoter activity in vasculature and base of trichomes of rosette leaf petiole, **(J)** transversal section of rosette leaf petiole showing signal in phloem (Ph), cortex (Cx), protoxylem (Px), and cambium (Ca). Scale bar in panels **B,C** is the same as in panel **D**. Arrows in panel **E** indicate shoot apices; a magnification of one apex is shown.

To gain insight into the function of *ANAC087*, its expression pattern was studied. Quantitative real-time RT-PCR using RNA from different tissues of wild-type Arabidopsis was carried out. The results show that the highest *ANAC087* transcript levels are found in root and rosette leaf followed by flower, stem, caulinar leaf, and silique, albeit at similar levels (transcript levels were normalized to rosette leaf = 1) (Figure 2A). These results are similar to those observed for *CmNACP1* (Ruiz-Medrano et al., 1999) in terms of the tissue showing the highest accumulation levels of both transcripts, lending support to the notion that these genes are orthologs. The expression pattern of the *ANAC087* gene was then studied with more detail through histochemical staining of tissues from four independent transgenic lines (L31, 150, L168, and L170) carrying the *ANAC087* promoter (3.0 kb upstream of the start codon, including the 5′UTR) fused to the *uidA-GFP* reporter gene, termed *PROM-ANAC087*. Strong GUS activity was observed in leaf vascular tissue in first-, second-, and third-order veins (Figure 2B), and also in trichomes of caulinar leaf petioles (Figure 2C). GUS signal was detected in stem sections (Figure 2D). Also, a strong signal was visible in shoot apices, which decreases sharply in underlying tissue (Figure 2E). This pattern is also reminiscent of other *NAC* genes that are expressed in the SAM and have a role in its maintenance, as in the case of Petunia *NAM* and Arabidopsis *CUC2*. Of note, GUS activity is evident in developing flowers, but absent in more mature open flowers, except in the papilla (Figure 2F). Histochemical GUS staining was also observed in mature seeds, which suggests a role in its development (Figure 2G). GUS activity was very strong in the main root and becomes weaker in lateral roots, albeit more pronounced activity was observed in sites of lateral root formation (Figure 2H). The staining pattern at the base of the leaf indicates expression of this promoter mostly in the vasculature, and also in the base of trichomes (Figure 2I). Finally, transverse section of rosette leaf petiole evinces expression in the vasculature, including phloem, protoxylem, cambium, and cortex (Figure 2J). In all, the expression pattern of *ANAC087* is reminiscent of *NAM* and *CUC2* in the shoot apex and of *CmNACP1* in the vascular tissue. This reinforces the notion that these genes may be, to some extent, functional homologs.

To determine the localization of the ANAC087 protein, a western blot was carried out with total and nuclear protein extracts from Arabidopsis rosette leaves and an antibody against ANAC087. The immunoblotting assay showed that this protein is present in total protein extracts and also in nuclear protein extracts (Supplementary Figure 2).

### *ANAC087* Overexpression Induces Leaf Senescence in Arabidopsis

**FIGURE 3:**
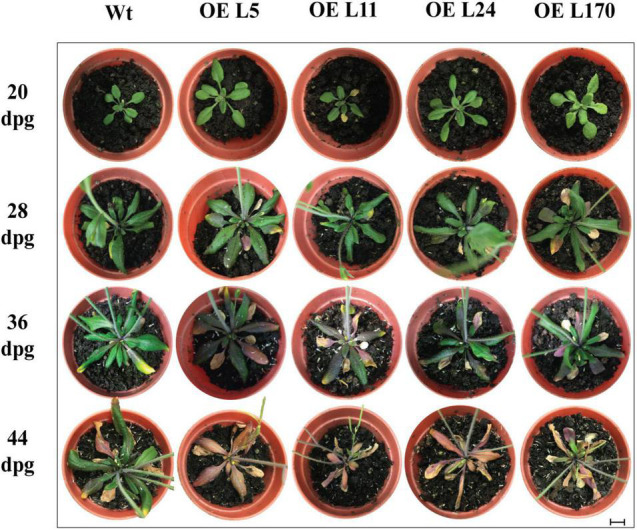
Senescence-associated phenotype induced by *ANAC087* overexpression in Arabidopsis rosettes. Wild-type plants and four different transgenic lines overexpressing *ANAC087* (OE L5, OE L11, OE L24, OE L70). Rosette leaves of overexpressing *ANAC087* lines presented anthocyanin accumulation at 36 dpg and early leaf senescence at the final time point (44 dpg). Photographs were taken at 20, 28, 36, and 44 days post-germination (dpg). Scale bar is 1 cm.

To infer the function of this gene, the phenotype of transgenic plants in which *ANAC087* was expressed constitutively was analyzed. *ANAC087* overexpression lines (*OE-ANAC087*: *OE* L5, *OE* L11, *OE* L24, and *OE* L170) were obtained and their development was evaluated at different time points. Images of plants at 20, 28, 36, and 44 days post-germination (dpg) were taken. We observed that at 36 dpg anthocyanin accumulation was stronger in *OE*-*ANAC087* lines relative to WT plants. Furthermore, at 44 dpg, we observed early leaf senescence and anthocyanin accumulation in *OE-ANAC087* plants (Figure 3). These observations confirm that *ANAC087* is a positive regulator of leaf senescence.

### Overexpression of *ANAC087* Induces Anthocyanin Accumulation, a Decrease in Chlorophyll Content, and Enhances Expression of Leaf Senescence-Related Genes

**FIGURE 4:**
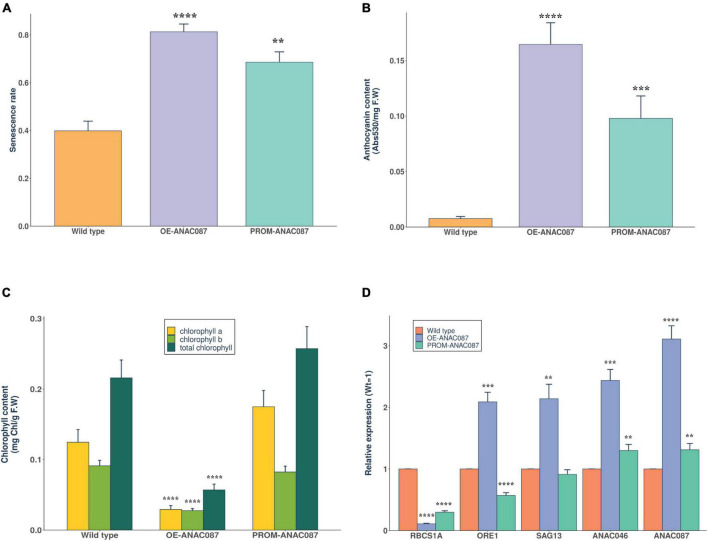
Breakdown of photosynthetic capacity and genetic regulation of senescence-related genes in *ANAC087* Arabidopsis transgenic lines. **(A)** The senescence rate of rosette leaves in *ANAC087* transgenic lines, calculation of senescence rate with the total number of photosynthetic and senescent leaves at the final stage of phenotypic analysis (44 dpg) in overexpression (*OE-ANAC087*) and promoter region (*PROM-ANAC087*). **(B)** Anthocyanin content expressed by the absorbance at 530 and normalized to leaf fresh weight (F.W) in wild-type, *OE-ANAC087*, and *PROM-ANAC087* plants. **(C)** Total chlorophyll, chlorophyll a, and chlorophyll b concentrations expressed by leaf fresh weight (F.W) in wild-type, *OE-ANAC087*, and *PROM-ANAC087* plants. **(D)** Quantification of relative expression of senescence downregulated genes (SDGs): *RBCS1A* and senescence-associated genes (SAGs) or upregulated during senescence: *ORE1, SAG13*, and *ANAC046* in comparison with *ANAC087* in all transgenic lines. Expression levels are reported with the Wt plants as 1 and normalized relative to *UBQ10* as endogenous gene. In all cases, the measurements for these experiments were determined in four different lines of each transgene with three technical replicates. Values are the means ± SEM (*n* = 12). The comparisons were made using Wilcoxon test, (**) *p* ≤ 0.01, (***) *p* ≤ 0.001, (****) *p* ≤ 0.0001.

Since the early leaf senescence phenotype was detected in most cases at 44 dpg stage, we collected rosette leaves at this stage to test different parameters that are related to the reduction in the photosynthetic capacity. First, we calculated the senescence rate of rosette leaves in all *ANAC087* lines. High senescence rate was obtained in *OE-ANAC087* and *PROM-ANAC087* lines, and there was a significant difference compared to wild-type plants (Figure 4A). Then, the anthocyanin content was measured as the absorbance at 530 nm and normalized to leaf fresh weight, and the *OE-ANAC087* and *PROM-ANAC087* lines showed high content levels of anthocyanins compared to wild-type plants (Figure 4B). Further, the total chlorophyll, chlorophyll a, and chlorophyll b concentrations were calculated in all transgenic lines. In *OE-ANAC087*, the concentrations of chlorophyll (total, a and b) decreased significantly compared to wild type. In *PROM-ANAC087* lines, no significant difference was observed compared to wild-type plants (Figure 4C). On the other hand, some studies report that photosynthesis is gradually inactivated during senescence and accompanied by degradation of chlorophyll and also anthocyanin accumulation (Bresson et al., 2018). Thus, the results in this work show that *ANAC087* expression is related to the process of leaf senescence, since *OE-ANAC087* lines showed higher anthocyanin accumulation and senescence rate relative to control plants. Also, chlorophyll concentrations (total, a, b) of *OE-ANAC087* lines were reduced. To analyze whether *ANAC087* is involved in senescence leaf process, we performed qRT-PCR for genes associated with senescence in Arabidopsis. The expression levels of *RBCS1A* from the downregulated gene set (SDGs) group during senescence were lower in *OE-ANAC087* followed by *PROM-ANAC087* lines than in wild-type plants. For genes that belong to senescence-associated genes (SAGs) group, we obtained the highest relative expression of *ORE1, SAG13*, and *ANAC046* in *OE-ANAC087* lines. Also, we observed that *ANAC087* expression levels were high and comparable to that one obtained in genes associated with senescence. The *PROM-ANAC087* lines presented higher levels of *ANAC046* and *ANAC087* than in wild-type plants (Figure 4D).

### Frequency of Aerial Rosettes and Increasing Branching Occurs in T1 and T2 *OE*- and *PROM-ANAC087* Plants

**FIGURE 5:**
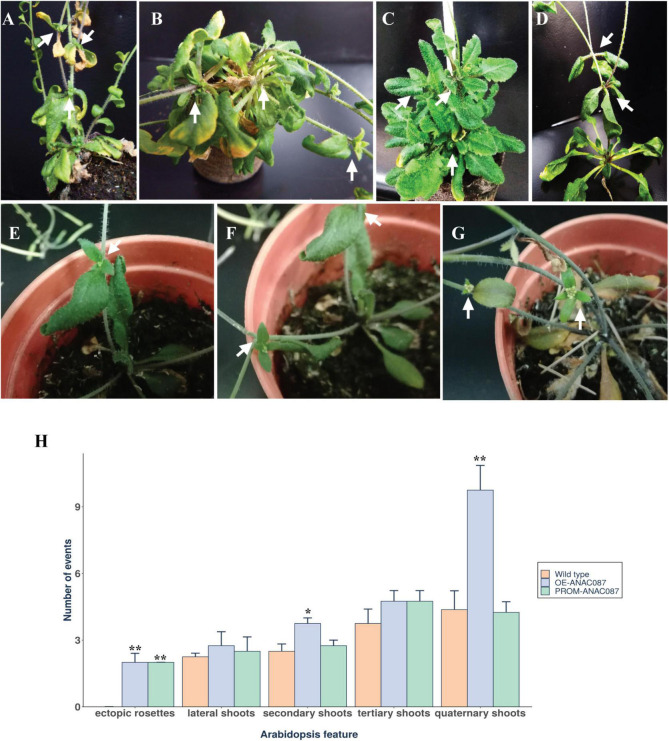
Aerial or ectopic rosette and branching phenotype induced in *OE*- and *PROM-ANAC087* transgenic lines. **(A–D)** Ectopic rosette growth along the main stem and lateral shoots from different *PROM-ANAC087* lines (T1 and T2). **(E–G)** The same phenotype was observed for *OE-ANAC087* T1 and T2 plants. Arrows indicate the position of aerial or ectopic rosettes along the stem and lateral shoots. **(H)** Ectopic rosettes, lateral, secondary, tertiary, and quaternary shoots (Arabidopsis features) were quantified in *OE* and *PROM-ANAC087* lines. Arabidopsis features were quantified in eight plants per line. Values are the means ± SEM. The comparisons were made using Wilcoxon test, (*) *p* ≤ 0.05, (**) *p* ≤ 0.01.

Another phenotype observed in different *PROM-ANAC087* (Figures 5A–D) lines and *OE-ANAC087* plants (Figures 5E–G) was the presence of aerial, or ectopic, rosettes. We observed the development of more than one ectopic rosette in each *OE*- and *PROM-ANAC087* plant. For *PROM-ANAC087* lines, this phenotype was more marked in *PROM*- than *OE-ANAC087* lines in T1 and T2 generations. Interestingly, leaves from ectopic rosettes showed early senescence compared to the main rosette leaves in *PROM-ANAC087* (Figure 5A). However, in a normal plant stage, senescence progression ends with the complete disintegration of leaf tissues, senescence occurring first in rosettes, which is not observed in *PROM-ANAC087* plants. In *OE-ANAC087* lines, we identified several ectopic rosettes that presented less foliage than *PROM-ANAC087* plants (Figures 5E–G). Furthermore, a significant increase in the branching of secondary and quaternary shoots was also observed in these transformed plants (Figure 5H). Thus, the *ANAC087* gene may also have an important role in regulating the Arabidopsis body plan. However, this was observed in T1 and T2 plants; T3 plants harboring either construct showed this phenotype at much lower frequencies, which suggests epigenetic silencing.

### Expression of the *ANAC087* Promoter Construct Enhances Accumulation of the Endogenous mRNA

**FIGURE 6:**
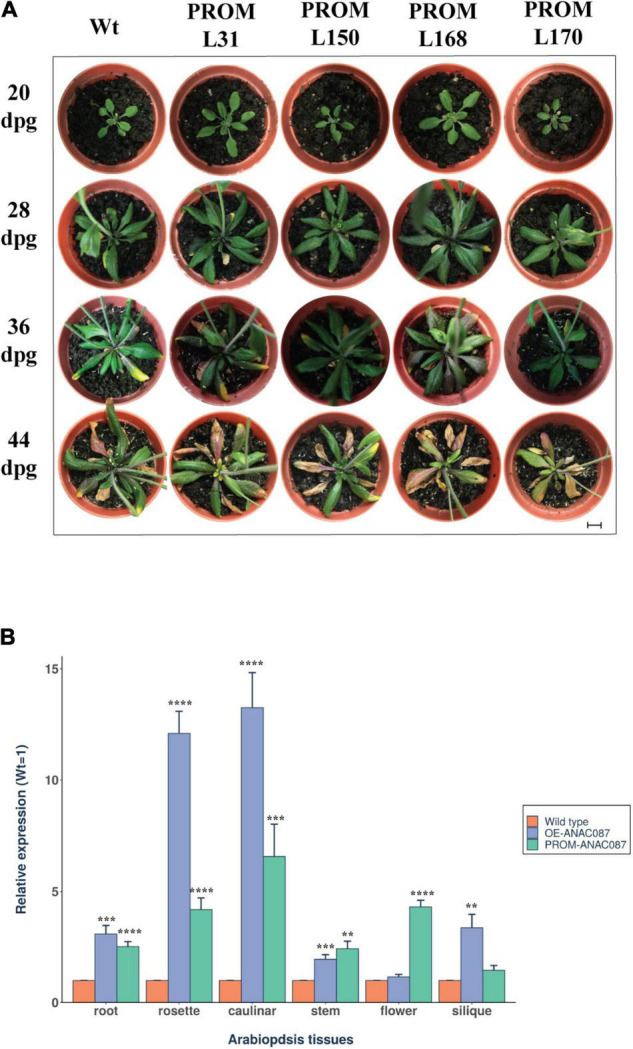
Phenotype induced by expression of *ANAC087* promoter and accumulation of the endogenous mRNA in *ANAC087* transgenic plants. **(A)** Transgenic lines directing GFP expression under *ANAC087* promoter region, 3.0 kb upstream sequence from the ORF (*PROM* L31, *PROM* L150, *PROM* L168, *PROM* L170). Photographs were taken at ages of 20, 28, 36, and 44 days post-germination (dpg). Scale bar is 1 cm. Anthocyanin accumulation and early leaf senescence were observed in *PROM*-*ANAC087* lines. **(B)** Quantification of *ANAC087* transcript in wild-type, *OE*-*ANAC087*, and *PROM*-*ANAC087* lines at the end of phenotypic analysis. The experiment was carried out with primers designed within the 3′end of the ORF, because this is highly variable among the NAC gene family members. Relative expression was determined by four different lines of each transgene with three technical replicates for each tissue (root, rosette leaf, caulinar leaf, stem, flower, and silique). Expression levels are reported with the wild-type plants as 1 and normalized relative to *UBQ10* as endogenous gene in all transgenic lines. The measurements were determined in four different lines of each transgene with three technical replicates. Values are the means ± SEM (*n* = 12). The comparisons were made using Wilcoxon test, (**) *p* ≤ 0.01, (***) *p* ≤ 0.001, (****) *p* ≤ 0.0001.

Intriguingly, in *PROM-ANAC087* lines, a phenotype similar to *OE-ANAC087* plants was observed after 44 dpg, in terms of anthocyanin accumulation, although early leaf senescence was not as pronounced as in overexpressing lines (Figure 6A). This suggested that the endogenous *ANAC087* gene was also overexpressed in the *PROM-ANAC087* lines. This in turn prompted us to assay the mRNA levels of *ANAC087* in the *OE* and *PROM* lines. Thus, qRT-PCR for this RNA was carried out in different tissues from four transgenic Arabidopsis lines for each transgene (*OE* and *PROM*) and compared to wild-type plants. In *OE-ANAC087* lines, the highest mRNA levels were found in caulinar and rosette leaves, followed by siliques, roots, stems, and flowers. In the case of *PROM-ANAC087* lines, increased accumulation of the endogenous *ANAC087* transcript occurred in all tissues (Figure 6B).

## Discussion

Several NAC transcription factors regulate senescence either positively or negatively. Others are involved in programmed cell death, a process related to senescence. The pumpkin *CmNACP1* mRNA which accumulates in mature, functional SE and moves long distance across a graft union into the shoot apex of a cucumber heterograft (Ruiz-Medrano et al., 1999) shows high similarity to the tomato senU5 NAC domain protein, involved in senescence, which suggest a dual role in both shoot apical maintenance and leaf senescence (John et al., 1997). Thus, the aim of this work was to determine, based on phylogenetic analysis, the closest homolog of CmNACP1 in Arabidopsis, to elucidate more clearly its function in a model system. The results indicate that these protein groups with NAC proteins that in general are positive regulators of leaf senescence, namely, *ANAC046* (At3g04060), *ANAC087* (At5g18270), *ANAC092* (At5g39610), *ANAC059* (At3g29035), and *ANAC080* (At5g07680) (Balazadeh et al., 2010; Oda-Yamamizo et al., 2016; Lee et al., 2017; Huysmans et al., 2018). The function of the other member of this clade, *ANAC100* (At5g61430), seems to be related only to leaf boundary formation (Laufs et al., 2004). *ANAC080* has a role in axillary meristem formation, lending support to the notion that members of this clade are also involved in developmental regulation (Raman et al., 2008). *ANAC046* and *ANAC087* were considered CmNACP1 closest homologs based on sequence similarity taking into account both only the NAC domain and the complete protein sequence and the corresponding *E*-value (not shown). *ANAC087* is expressed in leaf vascular tissue, which supports the notion that this gene and *CmNACP1* are also functionally related. Therefore, *ANAC087* was analyzed in more detail.

*ANAC087* is involved in developmental regulation judging from the phenotype observed in *OE*- and *PROM-ANAC087* plants, that is, an increased number of branches from inflorescence stems. Whereas both *CmNACP1* and *ANAC087* transcripts are expressed in the vascular tissue, the latter is not mobile in Arabidopsis (Thieme et al., 2015). This suggests that *ANAC087* has a cell autonomous function, in contrast to *CmNACP1* mRNA. However, CmNACP1 protein does not localize to mature SE, so its potential role as a long-range signal is limited to the transcript (Ruiz-Medrano et al., 1999). Immunolocalization of the ANAC087 protein in phloem tissue and also graft studies will determine whether this protein is also cell autonomous.

The phenotype of Arabidopsis *OE-ANAC087* is consistent with induction of leaf senescence. Additionally, *OE-ANAC087* (and also *PROM-ANAC087*; see below) plants show decreased chlorophyll content in leaves, and also increased anthocyanin content, both hallmarks of leaf senescence. This is further supported by the induction of senescence-related markers. However, the fact that this gene is expressed at very high levels in vascular tissue and the shoot apex points to additional functions, likely related to developmental regulation. On the other hand, the fact that this gene is not expressed in mature (and likely senescent) floral organs indicates that *ANAC087* induces cell death and senescence in a tissue-dependent manner.

Aerial rosettes are not usually observed in Arabidopsis *Col-0* or *Ler* ecotypes; however, they are a prominent feature in the *Sy-0* ecotype. The formation of these rosettes requires the combined action of the *FRIGIDA* (*FRI*) and *AERIAL ROSETTE 1* (*ART1*) genes to convert an axillary meristem into an ectopic rosette (Poduska et al., 2003). It was later demonstrated that *ART1* is an allele of the *HUA2* gene, which codes for a pre-mRNA processing factor, and is involved in the induction of floral repressor genes (Doyle et al., 2005). Thus, it is possible that the ectopic expression of *ANAC087* causes the dysregulation of *HUA2*, resulting in the emergence of aerial rosettes; if this is the case, then a similar dysregulation caused by ectopic expression of its ortholog in the *Sy-0* ecotype causes here the aerial rosette phenotype. It will be of interest to determine whether *ANAC087* directly regulates the expression of *HUA2* and/or the latter regulates the processing of the *ANAC087* mRNA. Unexpectedly, this phenotype was more marked in plants expressing the *PROM-ANAC087* construct, in that aerial rosettes were clearly separated from the primary rosettes. Again, it is worth mentioning that this construct includes the 5′UTR of this gene, so it is reasonable to assume that this portion of the transgene mRNA (i.e., coded by the *GFP-uidA* reporter gene) is responsible for such phenotype. No miRNAs are predicted in this region, so the underlying mechanism may not rely directly on PTGS. The fact that this construct also enhances the accumulation of *ANAC087* mRNA suggests that this overexpression causes the observed phenotype. Fusion of this region to other ORFs will help to resolve this question.

Another unexpected result from this work was the induction of senescence by both the *OE-ANAC087* and *PROM-ANAC087* constructs in transgenic Arabidopsis. The results shown herein indicate that this phenotype is caused in both cases by an increased accumulation of the *ANAC087* transcript and likely the protein as well. How is the *PROM-ANAC087* construct able to induce the corresponding endogenous gene remains to be determined; however, this is possibly the result of the 5′UTR sequence of this gene. As mentioned earlier, no microRNAs have been detected to target either the *ANAC087* ORF or the 5′UTR, and whereas the predicted secondary structure of this region would be stable, it is not clear how this could modulate the expression of the endogenous gene. More work is required to determine the mechanism underlying this potential regulation. In the *PROM-ANAC087* construct, the terminator used was *NOS*; these genetic elements, and, in particular, a strong polyA signal are the key for efficient accumulation of sRNAs when transgenes are expressed (de Felippes et al., 2020). This could negatively affect the expression of the transgene, although it is not clear how this could induce the accumulation of the endogenous *ANAC087* mRNA, considering that its accumulation is higher than WT when the promoter plus the 5′ UTR construct is expressed. The interactome of both *ANAC087* protein and RNA (including the 5′UTR) will afford information regarding the mechanisms through which these induce the observed phenotypes in Arabidopsis.

## Conclusion

*ANAC087* is one of the closest homologs of *CmNACP1* in Arabidopsis, although its RNA is not transported long distance, and has a role in promoting senescence of source leaves (rosette); its overexpression enhances this process in Arabidopsis. Furthermore, its ectopic expression correlates with the emergence of aerial rosettes, likely resulting from the induction of the *HUA2* gene, involved in flower induction. Finally, it is likely that the 5′UTR of the *ANAC087* gene is sufficient to induce the endogenous gene.

## Data Availability Statement

The original contributions presented in the study are included in the article/Supplementary Material, further inquiries can be directed to the corresponding authors.

## Author Contributions

BV-H, RR-M, and BX-C wrote the manuscript. BV-H performed most of the experiments. RR-M and BX-C obtained financial support for the work and designed the experimental plan. RR-M, BX-C, BC-P, and LN-M edited the manuscript. All authors agreed with the final version of the manuscript.

## Conflict of Interest

The authors declare that the research was conducted in the absence of any commercial or financial relationships that could be construed as a potential conflict of interest.

## Publisher’s Note

All claims expressed in this article are solely those of the authors and do not necessarily represent those of their affiliated organizations, or those of the publisher, the editors and the reviewers. Any product that may be evaluated in this article, or claim that may be made by its manufacturer, is not guaranteed or endorsed by the publisher.

## Footnotes

1
https://www.uniprot.org/

2
https://www.megasoftware.net/
