# Supplementary material for: The NAC Transcription Factor ANAC087 Induces Aerial Rosette Development and Leaf Senescence in Arabidopsis
Source: Front Plant Sci. 2022 Feb 23;13:818107. doi: 10.3389/fpls.2022.818107 (PMC8905224; doi:10.3389/fpls.2022.818107)
Supplement: Supplementary file 1 [file Data_Sheet_1.docx]

Supplementary Material

## Supplementary Figures and Tables


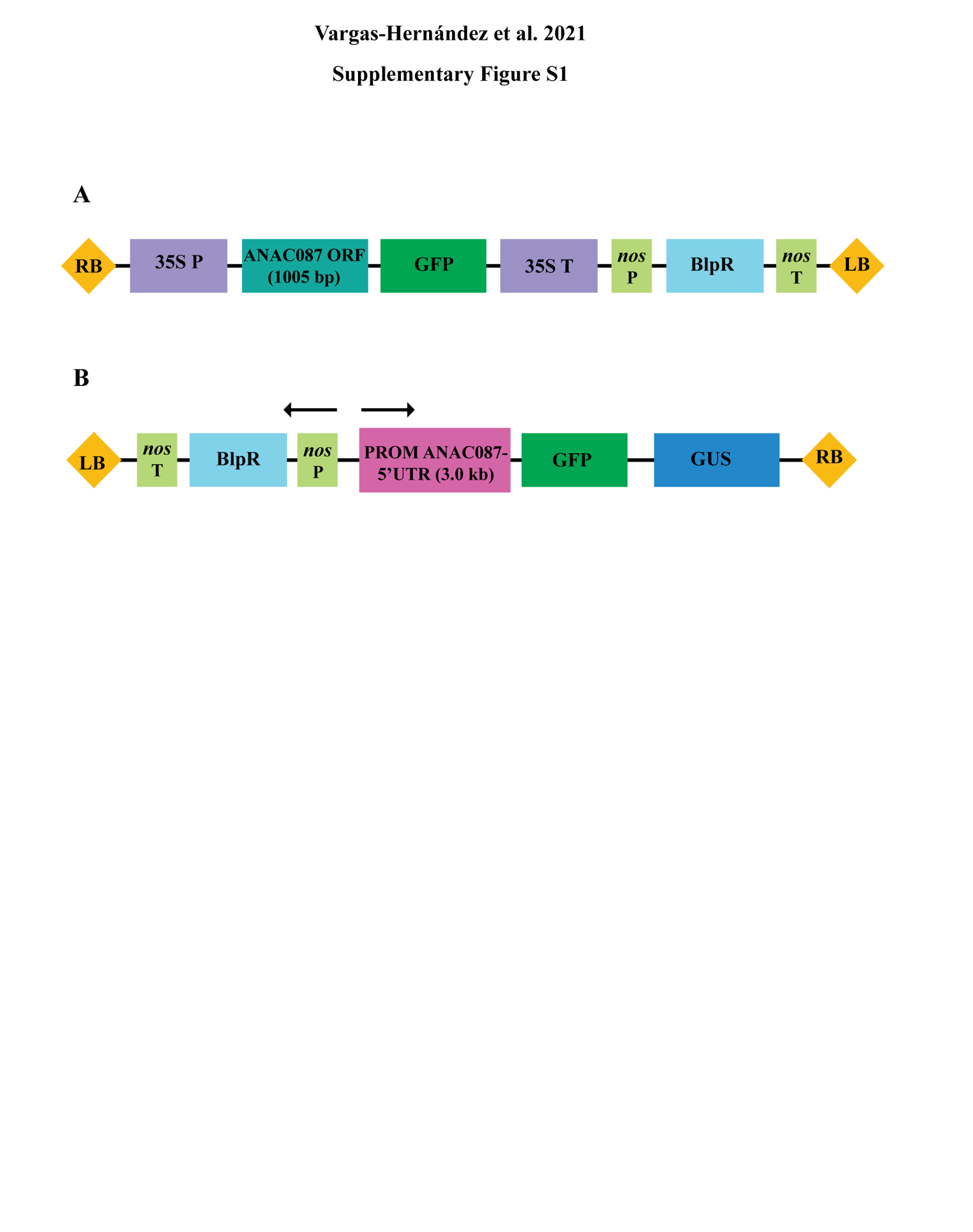


**Supplementary Figure S1.** Maps of *ANAC087* regions in Gateway binary vectors used for genetic analysis. **(A)** Construct for overexpression of *ANAC087* Open Reading Frame (ORF, 1005 bp) in pB7FWG2,0 binary vector. **(B)** The promoter region of *ANAC087* (3.0 kb) which includes the 5’ UTR cloned into pBGWFS7,0 Gateway vector. Left and right T-DNA borders (LB, RB), 35S promoter (35S P), 35S terminator (35S T), *nos* promoter (*nos* P), *nos* terminator (*nos* T), intron from Arabidopsis (intron), chloramphenicol resistance marker (CmR), Green-fluorescent protein (GFP), β-glucoronidase sequence (GUS), plant selectable marker that confers resistance to glufosinate ammonium (BlpR).


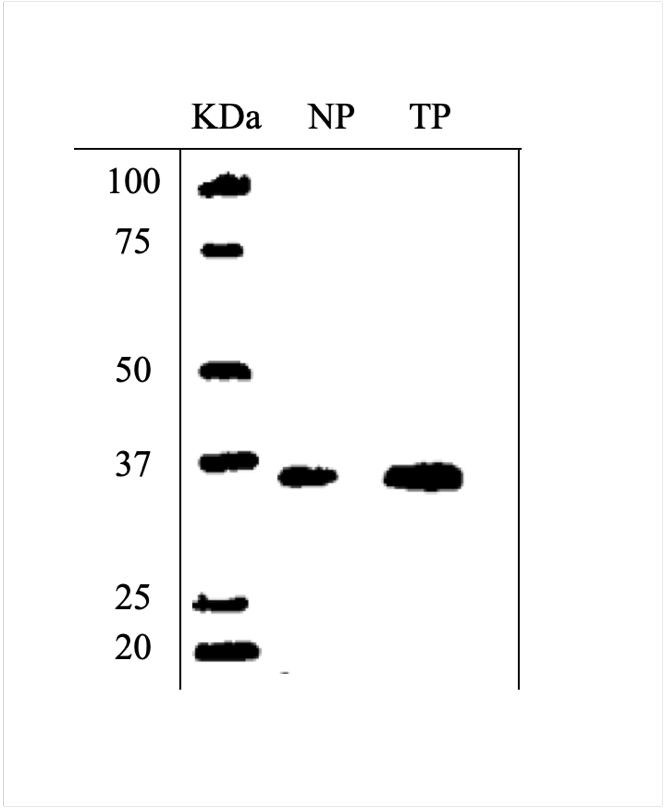


**Supplementary Figure S2.** ANAC087 protein is located in the nucleus. Total protein extract (TP) and nuclear protein extract (NP) were obtained from Arabidopsis rosette leaves, a western blot analysis was carried with an antibody against ANAC087 (1:2500) and a secondary antibody against rabbit IgG (1:10,000). Signal for ANAC087 protein (ca. 38 KDa) detection was observed in both protein extracts (NP and TP).

**Table S1.** Primers designed for *ANAC087* genetic constructs and used for qRT-PCR analysis

| **Primer name** | **Target** | **Sequence (5´→3´)** | **Size (bp)** | **Tm (°C)** | **Reference** |
| --- | --- | --- | --- | --- | --- |
| NAC87 ORF-F | ANAC087 Open Reading Frame | ATGGCGGTTGTGGTTGAAGAAGGTGTGGTG | 1005 | 68 | This work |
| NAC87 ORF-R |  | GAAGTCCCACAAGTCCCCCCTCAAGTCACA |  | 68 |  |
| NAC87 qPCR-F | 3´ end of ANAC087 ORF | TGATCAAGATGGAGCAATCACTTGTTAGTGT | 200 | 61 | This work |
| NAC87 qPCR-R |  | GAAGTCCCACAAGTCCCCCCTCAAGTCACA |  | 68 |  |
| NAC87 PROM-F | ANAC087 promoter and 5’UTR | CGACAAATCACCAATTTTCAACGGCGTG | 2988 | 64 | This work |
| NAC87 PROM-R |  | CACTTTATATGTTTTTCAAGTAATAAAGATTC |  | 53 |  |
| UBQ10 qPCR-F | Arabidopsis thaliana polyubiquitin 10 | CCCTCCACTTGGTCCTCAGGC | 195 | 64 | This work |
| UBQ10 qPCR-R |  | TTCTGCCATCCTCCAACTGC |  | 60 |  |
| RBCS1A qPCR-F | Arabidopsis ribulose bisphosphate carboxylase | ACCTTCCTGACCTTACCGATTCCG | 108 | 63 | Bresson et al., 2018 |
| RBCS1A qPCR-R |  | GGTACACAAATCCGTGCTCCAAC |  | 62 |  |
| ORE1 qPCR-F | NAC transcription factor ANAC092 | CTTACCATGGAAGGCTAAGATGGG | 114 | 60 | Bresson et al., 2018 |
| ORE1 qPCR-R |  | TTCCAATAACCGGCTTCTGTCG |  | 60 |  |
| SAG13 qPCR-F | Arabidopsis Senescence-associated gene 13 | AGGGAGCATCGTGCTCATATCC | 95 | 61 | Bresson et al., 2018 |
| SAG 13 qPCR-R |  | CCAGCTGATTCATGGCTCCTTTG |  | 62 |  |
| NAC46 qPCR-F | Arabidopsis NAC domain containing protein 46 | AGATCAGGAGGTGGTGGATTTG | 141 | 59 | This work |
| NAC46 qPCR-R |  | TTCTTGTTGAGGTCGGCTTG |  | 58 |  |
